# Bacterial counts in saliva of patients with percutaneous endoscopic gastrostomy: Cross-sectional research

**DOI:** 10.1016/j.jds.2025.06.027

**Published:** 2025-07-29

**Authors:** Madoka Funahara, Akira Imakiire, Ryuichiro Funahara, Sakiko Soutome, Yuki Sakamoto, Masahiro Umeda

**Affiliations:** a School of Oral Health Sciences, Faculty of Dentistry, Fukuoka, Japan; b Department of Oral Health, Nagasaki University Graduate School of Biomedical Sciences, Nagasaki, Japan; c Funahara Dental Clinic, Hyogo, Japan; d Department of Dentistry and Oral Surgery, Kansai Medical University Medical Center, Osaka, Japan; e Division of Research and Treatment for Oral and Maxillofacial Congenital Anomalies, School of Dentistry, Aichi Gakuin University, Nagoya, Japan

**Keywords:** Number of bacteria, Oral feeding, Percutaneous endoscopic gastrostomy, Saliva, Aspiration pneumonia

## Abstract

**Background/purpose:**

Dysphagia in older adults requiring long-term care often necessitates nutritional management via percutaneous endoscopic gastrostomy (PEG). Although PEG effectively prevents food aspiration, it does not mitigate the risk of silent saliva aspiration, leaving patients vulnerable to aspiration pneumonia. The purpose of this study was to evaluate the bacterial count in the saliva of PEG users.

**Materials and methods:**

This study included 13 PEG users and 13 oral feeders residing in a facility for individuals with disabilities. We investigated the participants’ age, sex, Eastern Cooperative Oncology Group performance status, oral wetness, Oral Hygiene Index Debris Index (OHI-DI), Winkel Tongue Coating Index (WTCI), number of teeth, and denture use. We also measured the number of bacteria in the saliva using the real-time polymerase chain reaction.

**Results:**

The study population comprised 12 men and 14 women. The oral feeding group had an average age of 61.9 years, whereas the PEG group had a lower average age of 50.6 years. The PEG group exhibited lower OHI-DI scores compared to the oral feeding group. However, the salivary bacterial count in the PEG group was over 10 times higher than that in oral feeders. Multivariate analysis revealed a higher salivary bacterial load in PEG users than in oral feeders, independent of oral hygiene indicators such as OHI-DI, oral wetness, and WTCI.

**Conclusion:**

PEG users demonstrated significantly higher salivary bacterial counts than oral feeders.

## Introduction

Aspiration pneumonia is a common and severe condition among older adults requiring nursing care, often resulting from a decline in swallowing function, aspiration of saliva containing pathogenic microorganisms, and reduced immune function.^1^ Aspiration pneumonia is also prevalent in individuals with impaired swallowing and those requiring ventilatory support, including ventilator-associated pneumonia (VAP) in patients receiving intubation. VAP is typically caused by aspiration of saliva or oral fluids contaminated with pathogens such as *Staphylococcus aureus*, *Streptococcus pneumoniae*, and gram-negative bacilli.^2^, ^3^, ^4^, ^5^

In Japan, percutaneous endoscopic gastrostomy (PEG) is frequently utilized to manage dysphagia in older adult patients, with the aim of preventing food aspiration. Although PEG prevents the aspiration of food, it does not mitigate the silent aspiration of saliva, leaving patients vulnerable to the risk of aspiration pneumonia. As oral feeding is not performed during PEG-assisted tube feeding, the amount of dental plaque is small, and the oral cavity appears clean. Previous studies have shown that changes in dietary consistency, such as transitioning from a normal to a liquid diet, increase the bacterial load in saliva among older individuals requiring care.^6^ Additionally, the absence of oral feeding in ventilated patients with oral intubation significantly elevates salivary bacterial counts due to the lack of swallowing and oral activity.^7^ Consuming regular food stimulates saliva secretion, cleanses the oral cavity through tongue movement and chewing, and facilitates the discharge of the food mass and saliva-containing bacteria into the digestive tract during swallowing. The growth of bacteria in the saliva is controlled by the self-cleaning function of the oral cavity. If PEG is used, the oral self-cleaning function diminishes, leading to an increase in the number of bacteria in saliva and a higher risk of aspiration pneumonia. However, studies comparing the bacterial count in the saliva of individuals fed orally versus those fed using PEG have not been conducted. This study aimed to investigate whether the bacteria count in the saliva differs between individuals fed orally and those fed using PEG.

## Materials and methods

### Study design and participants

This cross-sectional study targeted individuals with disabilities residing in facilities for persons with disabilities who require full support in daily life or special medical care. Participation was obtained with consent provided either directly by the participants or through their family members. Individuals unable to collect saliva due to difficulties, such as an inability to open their mouth, were excluded from the study.

Data collected included participants’ age, sex, Eastern Cooperative Oncology Group performance status (PS), oral wetness, Oral Hygiene Index-Debris Index (OHI-DI) scores,^8^ Winkel Tongue Coating Index (WTCI),^9^ and number of teeth. Oral wetness was measured using a dental mirror,^10^ with a score of 1 classified as xerostomia (−) and a score of 1–2 as xerostomia (+). The preliminary study included 30 participants, with the first participant enrolled on August 9, 2024.

### Measurement of salivary bacterial counts

The number of oral microorganisms in saliva was determined using the following procedure: A filter paper was placed under the participant's tongue for 5–10 s to absorb accumulated saliva, ensuring at least 1 cm from the tip of the filter paper was moistened. The moistened section (10 mm from the tip) was then placed in 500 μL of phosphate-buffered saline and allowed to stand for 30 min, after which deoxyribonucleic acid (DNA) was collected from the samples. Real-time polymerase chain reaction (PCR) was performed using specific primers, and the total bacterial count was calculated from a standard curve created using synthetic DNA. Primers and synthetic DNA were prepared were prepared following a previously described protocol.^11^ Real-time PCR conditions included thermal denaturation at 95 °C for 20 s, annealing at 62 °C for 90 s, and 40 cycles of DNA amplification. Fluorescent signals were detected after amplification under the following conditions: 95 °C for 15 s, 60 °C for 30 s, and 95 °C for 15 s. A melting curve was constructed to confirm the specificity of the amplified product.

### Statistical analysis

All statistical analyses were performed using SPSS v.26.0 (IBM Japan, Ltd., Tokyo, Japan). Factors related to the number of bacteria in saliva were analyzed using one-way analysis of variance (ANOVA) for categorical variables and Spearman's correlation coefficient for continuous variables, followed by multiple regression analysis. A two-tailed *P*-value of smaller than 0.05 was considered statistically significant.

## Results

A total of 26 participants were included in the study, with 13 participants each in the oral feeding and PEG groups (Table 1). The cohort comprised 12 men and 14 women. The mean age was 61.9 years in the oral feeding group and 50.6 years in the PEG group, indicating a younger demographic in the PEG group. In the oral feeding group, six individuals had an OHI-DI score of 2 or more, whereas all participants in the PEG group had scores of 0–1, indicating good oral hygiene.

**Table 1:** Characteristics of participants in the oral feeding and PRG groups.

Variable		Oral feeding group	PEG group	*P*-value
Age (years)		61.9 ± 10.9	50.6 ± 11.9	0.020
Sex	Man	5	7	0.695
	Woman	8	6	
PS	PS 0-1	0	0	0.160
	PS 3	12	8	
	PS 4	1	5	
Oral wetness	(−)/mild	13	11	0.480
	Moderate/severe	0	2	
OHI-DI	0–1	7	13	0.015
	2-	6	0	
WTCI	0–3	10	9	1.000
	≥4	3	4	
Number of teeth		21.8 ± 10.05	24.7 ± 7.85	0.430
Denture use	(−)	10	13	0.220
	(+)	3	0	

Total		13	13	–

Abbreviations PS: ECOG performance status, OHI-DI: oral hygiene index debris index, WTCI: winkel tongue coating index, PEG: percutaneous endoscopic gastrostomy.

**Figure 1:**
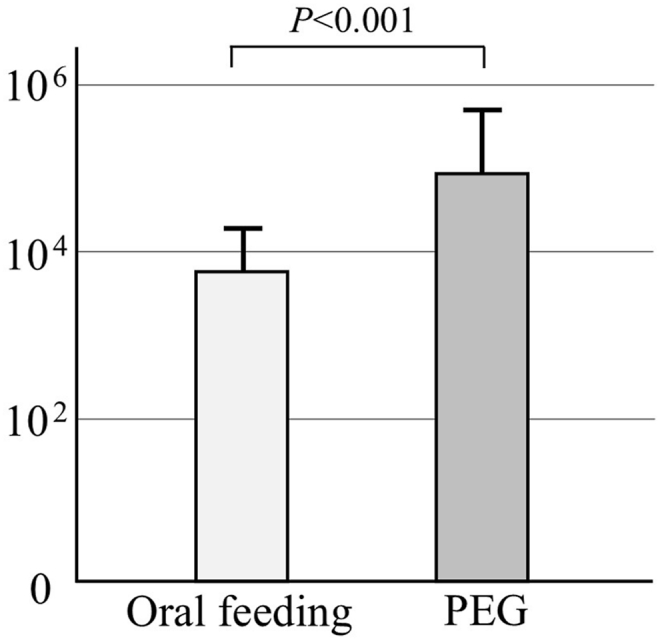
Total bacteria in saliva. The number of bacteria in the saliva of the PEG group increased more than tenfold compared to the oral feeding group.

The logarithmic bacterial count in the saliva was 3.76 in the oral feeding group and 4.94 in the PEG group, with significantly higher bacterial counts observed in the PEG group (Fig. 1).

We analyzed the factors related to the number of bacteria in saliva. In the univariate analysis, feeding status and age were significantly related to the number of bacteria; however, factors indicating oral hygiene status, such as oral dryness, OHI-DI, and WTCI, were not significantly related to the number of bacteria in saliva (Table 2). After performing multivariate analysis with factors that were significant in the univariate analysis as covariates, only feeding status was found to be associated with bacterial count. Participants in the PEG group had significantly higher bacterial counts compared to those in the oral feeding group (standardized coefficient: 0.674, 95 % confidence interval: 0.585–1.470, *P* < 0.001) (Table 3).

**Table 2:** Factors related to the number of bacterial in saliva (univariate analysis).

Variable		Logarithm of number of bacteria in saliva	*P*-value
i) Categorical data			
Sex	Man	4.47 ± 0.897	0.484
	Woman	4.25 ± 0.678	
PS	3	4.27 ± 0.789	0.387
	4	4.60 ± 0.753	
Oral wetness	(−)/mild	4.28 ± 0.773	0.148
	Moderate/severe	5.12 ± 0.297	
OHI-DI	0–1	4.46 ± 0.814	0.204
	2-	3.99 ± 0.554	
WTCI	0–3	4.27 ± 0.697	0.430
	≥4	4.55 ± 0.999	
Denture use	(−)	4.43 ± 0.769	0.168
	(+)	3.76 ± 0.691	
Feeding status	Oral feeding	3.76 ± 0.506	<0.001
	PEG	4.94 ± 0.497	

ii) Continuous data		Spearman's correlation coefficient	*P*-value

Age (years)		−0.519	0.007
Number of teeth		−0.056	0.786

Abbreviations: PS: performance status, OHI-DI: oral hygiene index, WTCI: winkel tongue coating index, PEG: percutaneous endoscopic gastrostomy.

**Table 3:** Factors related to the number of bacterial in saliva (multivariate analysis).

Variable	Unstandardized coefficient		Standardized coefficient	95 % confidence interval		P-value
	B	SE	β	Lower	Upper	
Age	−0.014	0.009	−0.224	−0.032	0.004	0.123
Feeding status (PEG/oral feeding)	1.028	0.214	0.674	0.585	1.47	<0.001

Adj. R^2^ = 0.610.

Abbreviations: PEG: percutaneous endoscopic gastrostomy, SE: standard error.

## Discussion

When oral feeding becomes difficult for various reasons, enteral nutrition may be chosen. Two widely used methods of enteral nutrition are nasogastric tube (NGT) and PEG.^12^ NGT is a method of enteral nutrition in which a tube is inserted from the nose into the stomach. This tube is often used for short-term nutritional support and is often used in hospitalized patients, those with reduced consciousness, or those with dysphagia. However, NGT is generally unsuitable for long-term use due to associated complications, including nasal and throat discomfort, an increased risk of infection, and a heightened risk of aspiration pneumonia. PEG is a common procedure used in patients requiring long-term tube feeding, in which a tube is inserted directly into the stomach through the abdominal wall using an endoscope. It is mainly used for patients with dysphagia due to cerebrovascular or neurodegenerative disease or for patients with chronic diseases who have difficulty taking food orally.^13^ The European Society for Clinical Nutrition and Metabolism guidelines (EPSEN guidelines) recommend NGT for temporary enteral nutrition lasting less than 4 weeks and PEG for cases lasting longer than 4 weeks.^14^ In Japan, PEG is often used for older adults who face long-term challenges with oral feeding due to diminished swallowing or cognitive function, with the aim of managing nutrition and preventing aspiration pneumonia. However, PEG is widely used for older adults who require nursing care, even if their swallowing function has not completely disappeared, to reduce the burden on caregivers.

Enteral nutrition allows for good nutritional management and reduces the risk of pneumonia caused by food aspiration; however, one of the main causes of aspiration pneumonia is the silent aspiration of saliva-containing pathogenic microorganisms, which is difficult to prevent even with enteral nutrition. In patients receiving enteral tube feeding, aspiration pneumonia is the most common cause of death,^15^^,^^16^ and the estimated incidence is reported to be 12–87 % in NGT^17^, ^18^, ^19^, ^20^, ^21^ and 9–52 % in PEG.^18^, ^19^, ^20^, ^21^ Tomioka et al.^22^ reported that the most common cause of death among patients who had been hospitalized for the treatment of pneumonia and who had received PEG was pneumonia, whereas Bourdel-Marchasson et al.^23^ reported that among older adult patients who required long-term care and who had received PEG, pulmonary complications occurred in 30 % of the control group and 39 % of the PEG group. Lin et al.^24^ and Chang et al.^25^ reported that the incidence of aspiration pneumonia was lower in the PEG group than in the NGT group. While there are reports that the risk of aspiration pneumonia is higher with NGT than with PEG, meta-analyses of pneumonia risk did not show that patients with NGT were at a higher risk than those with PEG. However, these results may be due to high levels of statistical heterogeneity between studies.^21^^,^^26^^,^^27^ NGT and PEG serve as effective methods for managing nutrition in patients with dysphagia, and they do not eliminate the risk of silent aspiration of saliva, a significant factor in the development of aspiration pneumonia. Preventing pneumonia remains challenging under these circumstances. Analyzing bacteria in saliva that contribute to pneumonia plays a crucial role in prevention. However, studies investigating the bacterial load in the saliva of patients undergoing enteral nutrition remain unavailable.

We examined factors related to the number of bacteria in the saliva of older adults requiring long-term care, people with disabilities, perioperative patients, and intubated patients.^7^^,^^8^^,^^11^^,^^28^, ^29^, ^30^, ^31^, ^32^ The results indicated that the number of bacteria was more closely related to eating conditions than oral hygiene. When food is consumed orally, the self-cleaning functions of saliva secretion, chewing, and swallowing work, and the number of bacteria in the saliva is maintained at a certain level. When the self-cleaning action of the mouth is reduced by endotracheal intubation, the number of bacteria in the saliva increases up to 100 times.^7^ Patients managed nutritionally through PEG do not engage in oral feeding, potentially leading to a reduction in the self-cleaning function of the mouth. Therefore, in this study, we examined patients receiving nutritional management via PEG and those receiving oral feeding using the number of bacteria in the saliva as an indicator. We found that the number of bacteria in the saliva was higher in patients receiving PEG than in those not receiving PEG.

When comparing the background factors of PEG and non-PEG recipients, PEG recipients were younger and had significantly better oral hygiene. With regard to oral hygiene, most PEG recipients had difficulty with self-care; therefore, their oral hygiene was checked by nursing staff at the facility three times a day, while most non-PEG recipients did not have their oral hygiene checked by nursing staff, suggesting that the oral hygiene of PEG recipients was better than that of non-PEG recipients. Although no caries or periodontal disease examinations were performed, the oral hygiene of the non-PEG recipients was not only poorer, but many of them also had more severe dental diseases, such as dental caries and periodontal disease. Nevertheless, the results showed that the number of bacteria in the saliva of the PEG recipients was significantly higher. This was thought to be due to the fact that the number of bacteria in the saliva was not greatly affected by the amount of dental plaque or calculus and that the self-cleaning function of the mouth had a greater effect.

We found that, in intubated patients with severely impaired oral function, as in patients undergoing PEG, brushing did not reduce the number of bacteria in the saliva or lower the risk of aspiration pneumonia. However, applying an antibacterial ointment to the mouth or wiping the mouth with povidone-iodine can suppress the increase in the number of bacteria in saliva for several hours.^11^^,^^33^^,^^34^ Further investigation is planned to investigate whether cleaning and wiping the mouth with povidone-iodine can also reduce the number of bacteria in saliva in patients undergoing PEG.

The study had several limitations. Firstly, the generalizability of the results remains uncertain because of the small sample size. Secondly, potential unknown bias may exist between PEG implementers and non-implementers, and despite performing multivariate analysis, this bias cannot be fully excluded. Thirdly, although the study quantified the total bacterial count using real-time PCR, bacterial species were not examined. Future research should focus on identifying microorganisms associated with pneumonia. Finally, pneumonia was not measured as an outcome, and only the number of bacteria in saliva was assessed. Thus, the direct relationship between an increase in bacterial count and the risk of aspiration pneumonia remains unclear. However, this is the first report to show that, even when brushing is encouraged, the number of bacteria in the saliva of people receiving PEG is higher than that in people who eat orally. Future studies should aim to increase the sample size and explore effective oral care methods to reduce bacterial counts in saliva and prevent aspiration pneumonia in patients receiving PEG.

In conclusion, the comparison of bacterial counts in the saliva of PEG recipients and oral feeders revealed significantly higher levels in PEG recipients, despite their good oral hygiene. These findings suggest that the oral self-cleaning plays a more critical role in controlling bacterial counts than oral hygiene alone. Future research should focus on developing oral care methods to prevent the rise in salivary bacterial counts and reduce the risk of aspiration pneumonia in patients receiving PEG.

## Ethics approval and consent to participate

This study conformed to the ethical guidelines of the Declaration of Helsinki and the Ethical Guidelines for Medical and Health Research Involving Human Subjects published by the Ministry of Health, Labour and Welfare of Japan. Ethical approval was obtained from the Institutional Review Board of The Japanese Society of Oral Care (no: E224002). The study protocol was registered in the University Hospital Medical Information Network (July 10, 2024; JPRN- UMIN000054916). All participants provided informed consent to participate in the study.

## Declaration of competing interest

The authors have no conflicts of interest relevant to this article.
